# Editorial: Advances in Childhood Sleep Assessment: Tools for Specific Populations

**DOI:** 10.3389/fpsyt.2021.647356

**Published:** 2021-03-05

**Authors:** Catherine Mary Hill, Carmen M. Schroder, Karen Spruyt

**Affiliations:** ^1^University Hospital Southampton NHS Foundation Trust, Southampton, United Kingdom; ^2^Hôpitaux Universitaires de Strasbourg, Strasbourg, France; ^3^Institut National de la Santé et de la Recherche Médicale (INSERM), Paris, France

**Keywords:** tool, child, sleep, questionnaire, developmental disability

In 2014 the Research Topic “Advances in Childhood Sleep Assessment: The Tool” was launched as a response to the publications “Development of pediatric sleep questionnaires as diagnostic or epidemiological tools: a brief review of dos and don'ts” (1) and “Pediatric Sleep Questionnaires as Diagnostic or Epidemiological Tools: A Review of Currently Available Instruments” (2). The field of pediatric sleep has greatly evolved since then and a real boost in new technologies and approaches is noted. This Special Issue demonstrates this boost with the publication of 14 studies dedicated to: children with neurodevelopmental conditions (autism spectrum disorder, Down syndrome, Angelman syndrome, Fragile X syndrome, bipolar disorder, *n* = 5), children with sleep disorders (*n* = 3), tools assessing sleep (*n* = 3), and three review papers on tools.

Sleep problems in children with developmental disabilities are reported to be common, yet their measurement is to this day challenging. In autism spectrum disorder, the validity of one brand of actigraphic measurement compared to polysomnography, the gold standard, was investigated by Yavuz-Kodat et al.. Authors concluded an acceptable clinical agreement between both tools to measure problematic sleeping in children with autism spectrum disorder. Similarly, the challenge to find an alternative to the gold standard is shown by Grantham-Hill et al.. Namely, in line with an increased demand to publish also negative results to improve science, the authors concluded that the psychometric properties of the screening questionnaire for obstructive sleep apnea in youth with Down syndrome were mediocre. This struggle for adequate tools is further demonstrated by multi-method approaches, which provide a broad range of sleep information but simultaneously are time and labor intensive, in an already challenged setting. A successful multi-method approach, reported by Trickett et al., assessing sleep quality and timing in children with Angelman syndrome demonstrated significant intra- and inter-individual variability. Such variability should inform us that assessment of and intervention for problematic sleeping in children with developmental disabilities needs a tailored approach. As in Dueck et al., multi-method approaches also show us what is feasible and what is not. In a Fragile X cohort, this study moreover focusses on sleep environment, biomarkers, and circadian rhythm data obtaining an all-round assessment, yet with necessary individual modifications during data collection. Lastly, Lopes et al. applying questionnaire and diary during each mood episode in youth with bipolar disorder showed that sleep complaints often occur during manic or depressive episodes, but equally well in both episodes. This study further highlighted that sleep problems may contribute to the maintenance of psychopathological symptomatology. Each of these aforementioned studies highlight the complexity associated with the measurement of sleep in youth with developmental disabilities but also the immense discrepancy between the need given the high prevalence and clinical demand vs. the scientific solution per the study limitations listed.

Systematic reviews appraising such diverse tools and assessment methodologies that fully capture problematic sleep behavior in children with developmental disabilities are therefore strongly encouraged. In this Special Issue, two narrative reviews and a systematic review are published. Bioulac et al. comprehensively discuss the subjective and objective methods to assess excessive daytime sleepiness in children with attention deficit hyperactivity disorder. Stubbs and Walters focus on questionnaires to assess pediatric restless legs syndrome, concluding that more studies are needed to validate a single question as well as existing adult versions of restless legs syndrome scales. Finally, in a systematic review, Sen and Spruyt take a broader scope discussing all available sleep questionnaires. That is, the original review of sleep questionnaires from 2011, which has led to the Special Issues on sleep tools supportively hosted by Frontiers, was updated to 2020 providing the pediatric sleep field a benchmark toward subjective sleep assessment. Nonetheless, each of the reviews emphasize that there is room for improvement in the development and psychometric evaluation of tools.

In typically developing children aged 8–9 years, Mazza et al. showed that self-report of sleep-wake patterns through use of a diary might be a means to obtain complementary subjective information. A more objective measure is local forehead and abdomen skin temperature, as Bach et al. reported from data collected in 6–12 year olds. This study offers a non-invasive measure of the sleep-wake cycle discussing the best and fewest number of sites for temperature measures. This proxy assessment of sleep via body temperature regulation in particular is potentially neglected in children with developmental disabilities. Smith et al. went one-step further in the discussion concerning accelerometery compared to the gold standard. Authors highlighted the interrelation of the device placement and the selected algorithm, demonstrating discrepancies in sensitivity and specificity toward sleep timing, quantity, and quality metrics.

The search for non-invasive methods and optimal bio-algorithms to assess sleep is progressively noted in the field, and also in this Special Issue. Measuring sleep disorders specifically was discussed in three papers. In the continued search to optimally appraise sleep disordered breathing Yanney et al. concluded that pulse transit time is more sensitive but less specific than oximetry. Galbraith et al. focused on annotating videos to assess chronic insomnia in children surviving brain tumors. That nocturnal behaviors in a naturalistic setting can be informative toward the diagnostic process is similarly explored by Gall et al.. Here, authors examined automatic 3D video analysis of children exhibiting rhythmic movement disorders to overcome laborious manual scoring of videos. Both studies address a clear need for standards in objective quantification of sleep behavior beyond the gold standard polysomnography or the widely used actigraphy, principally investigating sleep in the familiar sleep environment of a child.

We are thankful to the participating authors, the Frontiers team and its editors for contributions to a topic that warrants extra scientific attention. Special Issues like this provide a unique portal to publish papers on sleep methodology. Combined, the studies included indicate that we are embarking on a new era of sleep assessment in children with neurodevelopmental disorders. Indeed, the gold standard might not be the “holy grail” when assessing children with neurodevelopmental conditions. For these children, polysomnography is inherently challenging due to multiple electrode placement, an unfamiliar setting and unaccustomed procedures. Each child needs a personalized and resource intensive approach to successfully examine sleep through such standardized techniques. Furthermore, polysomnography is not the best tool for some sleep disorders which may be better assessed in the home setting using different technologies. Resource limitation also drives creative solutions to diagnosis and is necessary to meet the demand for sleep disorder diagnosis which in most countries significantly outstrips health service capacity. At such tipping points in the development of a specialist field, a creative process of trial and error is critical to advance practice. That is why hosting and contributing both positive and negative findings in Special Issues like this one are essential to advance the frontiers of sleep assessment in children.

## Author Contributions

KS drafted, CH and CS edited the editorial. All authors contributed to the article and approved the submitted version.

## Conflict of Interest

The authors declare that the research was conducted in the absence of any commercial or financial relationships that could be construed as a potential conflict of interest.
